# Supplementary material for: Predicting effective microRNA target sites in mammalian mRNAs
Source: eLife. 2015 Aug 12;4:e05005. doi: 10.7554/eLife.05005 (PMC4532895; doi:10.7554/eLife.05005)
Supplement: Figure 4—source data 1. — Using these coefficients and corresponding scaling factors (Table 3), context++ scores can be computed essentially as illustrated in Supplementary Figure 5 of Garcia et al. (2011). DOI: http://dx.doi.org/10.7554/eLife.05005.016 [file elife05005s001.docx]

**Figure 4–Source data 1.** Coefficients of the trained context++ model corresponding to each site type. Using these coefficients and corresponding scaling factors (Table 3), context++ scores can be computed essentially as illustrated in Supplementary Figure 5 of Garcia et al. (2011).

| **Feature** | **8mer** | **7mer-m8** | **7mer-A1** | **6mer** |
| --- | --- | --- | --- | --- |
| (Intercept) | –0.589 | –0.224 | –0.195 | –0.079 |
| TA_3UTR | 0.222 | 0.139 | 0.117 | 0.058 |
| SPS | 0.210 | 0.135 | 0.095 | 0.035 |
| sRNA1A | –0.018 | 0.010 | –0.025 | –0.002 |
| sRNA1C | –0.021 | 0.014 | –0.021 | 0.004 |
| sRNA1G | 0.060 | 0.062 | 0.030 | 0.018 |
| sRNA8A | 0.022 | 0.004 | –0.049 | –0.015 |
| sRNA8C | 0.012 | –0.031 | 0.033 | 0.016 |
| sRNA8G | 0.015 | –0.008 | –0.017 | 0.006 |
| Site8A | N/A | N/A | 0.000 | –0.002 |
| Site8C | N/A | N/A | 0.036 | 0.015 |
| Site8G | N/A | N/A | 0.015 | 0.012 |
| Local_AU | –0.254 | –0.177 | –0.075 | –0.040 |
| 3P_score | –0.040 | –0.055 | –0.060 | –0.024 |
| SA | –0.115 | –0.134 | –0.077 | –0.028 |
| Min_dist | 0.118 | 0.056 | 0.045 | 0.036 |
| Len_ORF | 0.205 | 0.100 | 0.063 | 0.029 |
| Len_3UTR | 0.310 | 0.154 | 0.129 | 0.045 |
| Off6m | –0.020 | –0.011 | –0.020 | –0.010 |
| ORF8m | –0.118 | –0.044 | –0.058 | –0.060 |
| P_CT_ | –0.103 | –0.048 | –0.048 | 0.005 |
